# Treatment of Dupuytren Disease With Injectable Collagenase in a Veteran Population: A Case Series at the Department of Veterans Affairs New Jersey Health Care System

**Published:** 2014-03-27

**Authors:** Aditya Sood, Paul J. Therattil, Angie M. Paik, Mary F. Simpson, Edward S. Lee

**Affiliations:** ^a^Division of Plastic and Reconstructive Surgery, Department of Surgery, New Jersey Medical School, Rutgers University, Newark, NJ; ^b^Department of Veterans Affairs New Jersey Health Care System, East Orange, NJ

**Keywords:** collagenase, Dupuytren disease, Xiaflex, palmar aponeurosis, flexion contracture

## Abstract

**Introduction:** Clinical trials seeking to establish long-term efficacy of injectable collagenase clostridium histolyticum for treatment of Dupuytren disease are ongoing. In this quality improvement study, the efficacy, recurrence rate, and complications of collagenase injection for Dupuytren disease are reviewed in a population of Veteran patients. **Materials and Methods:** A retrospective chart review was performed for patients who underwent treatment with injectable collagenase for Dupuytren disease from 2010 to 2013 at our regional Department of Veterans Affairs medical center. Data points of interest included the degree of joint contracture preoperatively, immediately after treatment, and at follow-up, complications, and patient satisfaction. **Results:** Sixteen patients received 27 injections (18 metacarpophalangeal and 9 proximal interphalangeal injections). The mean time of follow-up was 12.3 months. There was a 50% or greater reduction of the original extension deficit in 74.1% (n = 27) of the joints treated. Metacarpophalangeal joint recurrence was “high” (≥50°) in 0% (n = 18) of joints, and “low” (5°-50°) in 33.3% (n = 18) of joints with a mean follow-up of 12 months. Proximal interphalangeal joint recurrence was “high” (≥40°) in 18.5% (n = 9) of joints and “low” (5°-40°) in 7.4% (n = 9) of joints with a mean follow-up of 12.9 months. Minor complications were experienced in 93.8% (n = 16) of patients who underwent collagenase injection and included ecchymosis, skin laceration, injection-site swelling, injection-site hemorrhage, tenderness, and pruritus. Seventy-five percent (n = 12) of patients in our study reported they would undergo treatment with collagenase again. **Conclusions:** The case series presented demonstrates that injectable collagenase clostridium histolyticum produced a clinical success rate of 74.1% and is a safe method to treat Dupuytren disease.

Dupuytren disease (DD) is a fibroproliferative disorder in which pathologic collagen deposition in the palmar aponeurosis causes the formation of fibrous cords and flexion deformities.^1^^,^^2^ The metacarpophalangeal (MCP) or proximal interphalangeal (PIP) joints may be involved. The ring finger is most frequently involved, followed by the small finger, thumb, middle finger, and index finger.^3^^,^^4^ Although the pathophysiology of the disease is still being elucidated, current research indicates that the flexion contractures in DD are a result of myofibroblast contraction.^5^^-^7 There are many factors associated with DD including genetics, older age, male sex, occupation, trauma, alcoholism, smoking, diabetes, and possibly epilepsy.^8^^-^13

Patients are generally referred for treatment of DD when joint contracture progressively worsens or if the patient begins to experience significant disability of the hand.^3^ Surgery is the most common treatment, and contracture of the MCP joint of more than 30° or any degree of PIP joint contracture are indications for surgery.^14^ There are several surgical options for DD, including various types of fasciotomy and fasciectomy with limited fasciectomy as the criterion standard.^15^ Reported success rates for limited fasciectomy range from 53% to 79% depending on the degree of contracture.^16^^,^^17^ Up to 15.7% of patients who undergo fasciectomy, however, reportedly experience major complications including digital nerve (3.4%) or digital artery injury (2.0%), infection (2.4%), hematoma (2.1%), or complex regional pain syndrome (5.5%). Digital nerve and artery injuries are 10 times more common in those with recurrent DD.^18^

Until recently, medical treatment of DD has had limited clinical success.^19^^,^^20^ In 2010, the Food and Drug Administration approved the use of collagenase clostridium histolyticum, an enzymatic mixture of proteinases that is harvested and purified from bacterial cultures and marketed under the trade name Xiaflex (Auxilium Pharmaceuticals, Malvern, Pennsylvania). Collagenase can be injected directly into Dupuytren cords to digest enzymatically the collagen triple helix and decrease the proliferation of fibroblasts.^21^^,^^22^ Preliminary studies have shown promising results where 64% of joints that received collagenase injection had a reduction in contracture within 0° to 5° of full extension without serious complications.^23^

Collagenase has proven to be a noninvasive and safe alternative to surgical release of contracture; however, the outcomes, rate of recurrence, and high cost have led surgeons to wonder whether collagenase is a sustainable treatment for DD. As collagenase is still a relatively new treatment option on the market, literature on clinical success is somewhat limited. The goals of our study were to determine the efficacy, recurrence rate, and complication risks associated with treatment of DD using collagenase in a unique population of Veteran patients. This population of patients presented with an average of 48.8 months duration prior to seeking treatment, and 56.25% (or 9 of 16) patients had multiple sites and cords involved prior to treatment.

## MATERIALS AND METHODS

### Study population, diagnosis, and monitoring

Retrospective chart review was performed for 16 patients diagnosed with DD, who were treated with injectable collagenase between 2010 and 2013 by the senior author. There were a total of 29 injections (18 MP, 9 PIP, and 2 thumb web space) of which the thumb web space injections were excluded in this study. The patients in this case series presented to our regional Veterans Affairs medical center for evaluation of joint contracture of the hand. This study was conducted as a quality assurance/quality improvement. Informed consent including any necessary HIPAA consent was obtained from each patient, and institutional review board approval was not deemed necessary for this quality assurance/quality improvement study. Patients were interviewed and examined at the time of presentation. Clinical diagnosis was confirmed by palpating nodules or cords in the palm, or by assessing finger bending in affected joints. The degree of fixed-flexion contracture was measured with a goniometer after fingers were extended to a firm end point. Subjective evaluation of the disease severity was also graded for each patient by the senior author as mild, moderate, or severe. Passive and active range of motion was assessed after treatment. Joints were monitored for contracture recurrence throughout the follow-up time period. Those joints that had recurrence of 50° or more in MCP joints and 40° or more in PIP joints were considered “high” degree. Those joints that had recurrence from 5° to 50° in MCP joints and 5° to 40° in PIP joints were deemed “low” degree.

### Treatment

Those patients with palpable cords causing dysfunction at the time of examination were deemed eligible for treatment with collagenase injection. At the time of treatment, cords were palpated and injected directly with 0.58 mg of collagenase reconstituted in sterile diluent. Patients returned to the plastic surgery clinic the following day for passive extension manipulation in attempt to break the cord. Patients were placed in an extension finger splint for 3 weeks postinjection worn at nighttime only. If the contracture remained after 3 weeks, or the next follow-up date, and a cord was still palpable, injection and manipulation were offered up to 3 times total per cord. Clinical observation of patients was continued until the contracture was no longer palpable, the patient was satisfied with the result, or the patient was lost to follow-up. The degree of improvement of the contracture was also graded by the attending physician at each patient visit as “no change,” “satisfactory,” or “outstanding.” Patients were surveyed at the end of their treatment to see whether they would undergo treatment with collagenase again to assess overall satisfaction.

### Statistical analysis

Fischer's exact test was used to determine association between categorical variables. Independent samples *t* test was used to determine association with reduction in contracture. A significance level of *P* < 0.05 was used.

## RESULTS

Between 2010 and 2013, a total of 16 patients with a mean age of 69.9 years were treated with collagenase injection for DD at our Veterans Affairs medical center. The patient profile of our case series (n = 16) is shown in Table 1. It is important to note that the mean duration of disease prior to presentation for treatment was 48.8 months with a mean flexion contracture in the affected digits at presentation of 48.3° for MCP joints (n = 18) and 77.8° for PIP joints (n = 9).

Thirteen MCP and 7 PIP joints were treated with 27 total injections. Patients received a mean of 1.6 injections each. Treatment outcomes by finger are shown in Table 2. General improvement of fixed flexion contracture based on physician rating (those rated as either “satisfactory” or “outstanding”) was seen in 87.5% (n = 16) of patients. A reduction of 50% or more of contracture was achieved in 74.1% (n = 27) of joints treated. There was no statistically significant difference in clinical improvement or reduction of contracture between the ring and small finger. There was no statistically significant difference in clinical improvement or reduction of contracture between those patients who had 1 joint affected and those patients who had more than 1 joint affected (Table 3). Of all joint treatments with collagenase, 74.1% (n = 27) achieved objective clinical success as noted earlier, including 83.3% of MCP joints (n = 18) and 55.6% of PIP joints (n = 9) (Table 4). The mean reduction in flexion contracture was 35.7° in affected MCP joints (n = 18) versus 29.4° in PIP joints (n = 9). The mean reduction in flexion contracture was 35.4° in “mild/moderate” joints versus 31.4° in “severe” joints (Table 5). Although not statistically significant, those with fewer joints affected, MCP joint disease, and mild/moderate disease had higher rates of clinical improvement and greater improvement of the degree of their contracture.

Of all joints treated with collagenase injection, none had “high” (≥50°) recurrence in MCP joints, and 33.3% had “low” (5°-50°) recurrence at a mean follow-up of 12 months (range: 2-22 months) (Table 6). Similarly, 18.5% had “high” (≥40°) recurrence in PIP joints and 7.4% had “low” (5°-40°) recurrence at a mean follow-up of 12.9 months (range: 7-25 months).

### Safety

The complication rate in our series of patients was 93.8%, with 15 patients experiencing at least one treatment-related adverse event. Most of these adverse events included injection site ecchymosis/swelling, and 6 of the 27 injected sites (or 37.5%) demonstrated a skin tear after postinjection straightening, all of which healed with no further complications. No patients experienced severe adverse events including tendon rupture or prolonged injection site symptoms greater than 2 weeks. Complications experienced with collagenase injection in our series are reviewed in Table 7.

### Satisfaction

Seventy-five percent of patients in our study reported that they would undergo treatment with collagenase again.

## DISCUSSION

Our study was a single surgeon's experience with collagenase injection for 16 patients with DD. One of the goals of the study was to determine the rate of clinical success following treatment. To standardize improvement based on the starting degree of contracture, a reduction in contracture of 50% or more was used as the criteria for objective clinical improvement. In our study, we found that 74.1% (n = 27) of joints treated had a clinical improvement from the original extension deficit—a slightly larger proportion of patients than quoted by previous studies. Hurst et al^23^ reported the first results from the Collagenase Option for Reduction of Dupuytren (CORD) I study: a phase 3 randomized controlled trial comparing collagenase injection to placebo to treat DD. In 308 patients, 64% of joints that received collagenase injection had a reduction in contracture within 0° to 5° of full extension compared to 6.8% of those that received placebo. In the 66 patients in the CORD II trial, 44.4% of joints that received collagenase injection met the primary end point of a reduction in contracture to 0° to 5° of normal versus 4.8% of those that received placebo.^24^ This discrepancy between our findings and those of the CORD trials are most likely a result of our criteria for clinical improvement, which differed from those set by the CORD studies.

Another goal of our study was to determine whether there was a discernible difference in clinical outcome between MCP and PIP joint contractures. Of those joints with MCP contracture, 83.3% (n = 18) showed clinical improvement compared to only 55.6% (n = 9) of PIP contractures. More specifically, 92.3% (n = 13) of affected MCP joints achieved reduction to less than 30° of contracture by the last follow-up visit, which would preclude the indication for surgical intervention. This finding is concordant with results of the CORD I study where more MCP joints met the primary endpoint (76.7%) than PIP joints (40%).^23^

While it is established that collagenase is able to attain significant short-term improvements in contractures with a low incidence of adverse events, the long-term recurrence rate of DD with collagenase treatment is only now being elucidated. Long-term outcomes from the CORD trials revealed that rate of contracture recurrence after collagenase injection was 27% for MCP joints and 56% for PIP joints at 3 years. Twenty-three percent had “severe” disease.^25^ In our case series, 59.3% (n = 16) of patients experienced DD recurrence at a mean of 12.3 months follow-up. Fifty percent of MCP joints (n = 16) experienced DD recurrence at a mean follow-up of 12 months. Seventy-eight percent (n = 9) of PIP joints experienced DD recurrence at a mean follow-up of 12.9 months. Of “mild” and “moderate” contractures, 45.5% (n = 11) recurred. Meanwhile, 68.8% (n = 16) of “severe” contractures recurred. Further follow-up time is necessary to compare our outcomes with those of long-term collagenase studies and other less-invasive procedures, like percutaneous aponeurotomy, but we hypothesize that the high rate of recurrence in our series may be due to the high frequency of “severe” disease.

A final goal of the study was to gauge overall patient satisfaction and frequency of complication from collagenase use. Patients treated with collagenase had an overall favorable opinion of their results with 75% of patients in our study reporting they would undergo treatment with collagenase again. In our series of patients, we report a total complication rate of 93.8% (n = 16); however, there were no serious complications. Complications included ecchymosis, skin laceration, injection site swelling and hemorrhage, pruritus, and tenderness. Although 96.6% of patients in the collagenase group experienced adverse events in the CORD I trial, a vast majority of these were mild to moderate in intensity and resolved within 10 days without treatment.^23^ In the CORD I and II trials, there were 5 serious adverse events related to treatment: 2 tendon ruptures, 1 flexion pulley rupture, 1 case of complex regional pain syndrome, and a proliferation of a Dupuytren cord with sensory changes.^23^^,^^24^ With reporting of additional cases and complications, we will be able to better define the risks of collagenase injections. The results of our series seem to corroborate that collagenase is a safe intervention with low risk of serious complications, especially when compared with other surgical options available.

## CONCLUSIONS

Pending outcomes on long-term safety and recurrence, collagenase may come to play a more prominent role in the treatment of DD, especially for recurrent DD in patients who are reluctant or unable to undergo surgery. However, additional research is needed regarding the cost-effectiveness of collagenase injection, and whether certain subpopulations of those with DD fair better with collagenase treatment. Nonetheless, the results of our case series are consistent with those of previous studies and add to the growing literature showing that injectable collagenase clostridium histolyticum appears to be a safe and effective treatment for patients with DD, and contribute to a high degree of patient satisfaction with their treatment outcomes, including those from our regional Veterans Affairs medical center.

## Figures and Tables

**Table 1:** Baseline patient characteristics

Characteristic	
Age, y	
Mean (SD)	69.9 (6.8)
Range	62-86
Male, n (%)	16 (100.0)
White, non-Hispanic ethnicity, n (%)	15 (93.8)
Hand with 1 or more contractures, n (%)	
Left	5 (31.3)
Right	4 (25.0)
Both	7 (43.8)
Affected joints per affected hand, n	
Mean (SD)	2.0 (1.0)
Range	1-4
Affected MCP joints per patient, n	
Mean (SD)	1.2 (0.7)
Range	0-3
Affected PIP joints per patient, n	
Mean (SD)	0.5 (0.5)
Range	0-2
Family history of Dupuytren contracture, n (%)	2 (12.5)
History of risk factors and associated conditions, n (%)	
Diabetes	6 (37.5)
Current tobacco use	6 (37.5)
Previous tobacco use	6 (37.5)
Current alcohol use	5 (31.3)
Hand trauma	3 (18.8)
Knuckle pads	2 (12.5)
Vibration exposure	1 (6.3)
Epilepsy	0 (0.0)
Ledderhose disease	0 (0.0)
Peyronie disease	0 (0.0)
Age at diagnosis, y	
Mean (SD)	60.8 (11.7)
Disease first detected by, n (%)	
Finger bending	12 (75.0)
Nodules	3 (18.8)
Pain	6 (37.5)
Duration of symptoms when medical treatment first sought, mo	
Mean (SD)	48.8 (73.9)
Median (range)	24.0 (6-312)
Prior treatment for Dupuytren contracture, n (%)	
None	12 (75.0)
Surgery	3 (18.8)
Injection	2 (12.5)
Hand therapy	1 (6.3)
Other	0 (0.0)
Patient rating of disease severity at baseline, n (%)	
Mild	4 (25.0)
Moderate	5 (31.3)
Severe	7 (43.8)
Physician rating of disease severity, n (%)	
Mild	4 (14.8)
Moderate	7 (25.9)
Severe	16 (59.3)
Baseline contracture of treated joints (degrees)	
MCP contracture, mean (SD)	48.3 (24.7)
PIP contracture, mean (SD)	77.8 (25.9)

**Table 2:** Comparison of treatment outcomes by finger*

Outcome	
Clinical improvement by finger, n (%)	
Small	5 (71.4)
Ring	9 (64.2)
Middle	4 (66.7)
Index	0 (0.0)

*Clinical improvement was defined as reduction of the contracture by 50% or more from baseline.

**Table 3:** Comparison of treatment outcomes by number of joints affected

	1 joint affected	>1 joint affected	*P*
Clinical improvement, n (%)	15 (83.3)	5 (55.6)	0.68
Mean change in contracture from baseline (degrees)	39.4 ± 27.0	31.2 ± 23.3	0.43

**Table 4:** Comparison of treatment outcomes by joint

	MCP joint	PIP joint	*P*
Clinical improvement, n (%)	15 (83.3)	5 (55.6)	0.18
Mean change in contracture from baseline (degrees)	35.7 ± 23.6	29.4 ± 26.4	0.54

**Table 5:** Comparison of treatment outcomes by contracture severity

	Mild/Moderate	Severe	*P*
Clinical improvement, n (%)	13 (86.7)	7 (58.3)	0.19
Mean change in contracture from baseline (degrees)	35.4 ± 24.3	31.4 ± 25.0	0.68

**Table 6:** Recurrence of contracture

	Recurrence, n (%)
MCP—High (≥50°)	0 (0.0)
MCP—Low (5°-50°)	9 (33.3)
PIP—High (≥40°)	5 (18.5)
PIP—Low (5°-40°)	2 (7.4)

**Table 7:** Treatment-related adverse events

Adverse events	n (%)
Patients with ≥ 1 treatment-related adverse events, n (%)	15 (93.8)
Ecchymosis	7 (43.8)
Skin laceration	6 (37.5)
Injection site swelling	4 (25)
Injection site hemorrhage	1 (6.3)
Pruritus	1 (6.3)
Tenderness	1 (6.3)
